# Hepatic Benign Cystic Mesothelioma in Adults: A Case Report of a Rare Hepatic Cyst

**DOI:** 10.7759/cureus.51832

**Published:** 2024-01-08

**Authors:** Abdullah Mohamed, Sherif Elsherif, Raafat Makary

**Affiliations:** 1 Pathology and Laboratory Medicine, University of Florida College of Medicine – Jacksonville, Jacksonville, USA; 2 Radiology, University of Florida College of Medicine – Jacksonville, Jacksonville, USA; 3 Neuropathology, University of Florida, Jacksonville, USA

**Keywords:** cystic lesion, primary liver lesion, peritoneal inclusion cyst, hepatic cyst, benign cystic mesothelioma

## Abstract

Benign cystic mesothelioma (BCM), also known as peritoneal inclusion cyst, is a benign mesothelial lined cystic lesion, nearly always described in the pelvis of adult females. The hepatic location of BCM is rarely reported in the literature.

We report a case of hepatic benign cysts in a 65-year-old woman that was incidentally discovered by imaging studies 12 years ago as a small cyst. Recently, the patient started having abdominal discomfort, distension, and anxiety. A CT scan revealed two low-density fluid-filled cystic lesions, the largest in the caudate lobe measuring up to 10.7 cm and causing a mass effect on hepatic veins and inferior vena cava. Laparoscopic marsupialization of the large liver cyst was done without complications. On gross examination, the collapsed cyst wall was a thin partly translucent pale tan to pink membranous structure with fine vascularity. No discrete nodularity or solid lesion was identified. Microscopic examination showed a thin fibro-connective wall lined by a single layer of flat cuboidal cells with no cellular atypia. The cyst lining showed characteristic calretinin-positive immunohistochemical reactivity for mesothelium, supporting the diagnosis of BCM. Hepatic BCM is among a broad differential spectrum of cystic liver lesions ranging from developmental, reactive, inflammatory, and infectious lesions, benign to premalignant or frankly malignant neoplasms with different treatment strategies. Although BCM is the rarest among the long list of differential diagnoses of hepatic cysts, its identification in this rarely reported location is essential to avoid aggressive surgical treatment.

## Introduction

Benign cystic mesothelioma (BCM), also known as benign inclusion cyst, occurs predominantly in the peritoneum mainly in the pelvis of adult females, and is very rarely reported in the liver. Plaut reported BCM for the first time in 1928, and later, in 1979, Menemeyer and Smith provided a description of the lesion [1]. Despite the unclear pathogenesis, it is considered in some studies as a reactive reaction of the peritoneum to previous intra-abdominal surgeries or inflammation. Other studies suggested benign neoplasm from its tendency to recur and rare malignant transformation [1]. It is usually asymptomatic and is often discovered incidentally during imaging studies performed for other reasons or can cause symptoms such as abdominal pain and distension or mass effect in involved or adjacent organs. We present a rarely reported site for BCM in the liver with clinical presentation, imaging studies, and pathological description with a brief literature review.

## Case presentation

**Figure 1:**
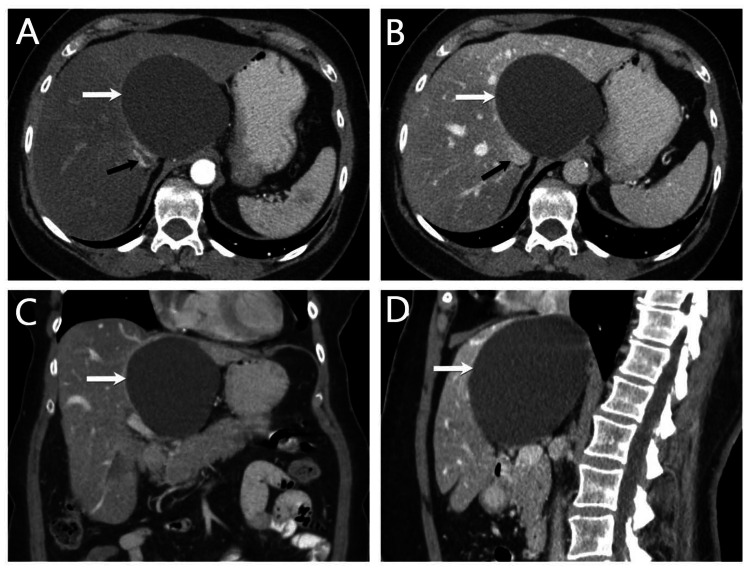
Abdominal CT scan Axial arterial phase (A) axial venous phase, (B) coronal venous, (C) and sagittal venous, and (D) CT images of the abdomen show a large circumscribed hypoattenuating cystic structure (white arrows) within the caudate lobe of the liver measuring up to 10.7 cm, causing a significant mass effect on the surrounding structures, including the IVC (black arrow) and hepatic veins. There is a background of moderate hepatic steatosis. No vascular invasion or lymphadenopathy.

A 65-year-old woman presented to the outpatient clinic complaining of abdominal discomfort, fatigue, and sleep disturbance. She denied abdominal pain, vomiting, or diarrhea. She has a past medical history of small liver cysts incidentally discovered by imaging studies 12 years ago during evaluation for hepatosteatosis, which did not raise concern for surgical treatment at that time. Recently the patient underwent left sparing mastectomy for moderately differentiated ductal carcinoma in situ with microinvasion diagnosed from stereotactic guided breast biopsy five to six months ago. On physical examination, the patient was hemodynamically stable with a temperature of 36.8°C, and blood pressure of 147/91 mmHg. Abdominal examination was unremarkable. Abdominal CT scan showed hepatomegaly, steatosis, and two low-density fluid-filled intrahepatic cystic lesions, the largest in the caudate lobe up to 10.7 cm causing mass effect compression of hepatic veins and inferior vena cava (Figure 1). Compared to her last CT scan, which was one year ago, the large cyst in the caudate lobe was 7.5 x 6.7 cm. The cyst characteristics in CT favored benign cystic lesions with differential including a simple hepatic cyst and biliary hamartoma.

Serum carcinoembryonic antigen was elevated at 4.8 (normal < 2.5 in non-smokers) and serum carbonic anhydrase was normal. The elevated serum level of CEA, in the context of the clinical history and CT findings, was not raising strong concern for malignancy as it can be a non-specific indicator seen in a wide range of lesions, including benign, inflammatory, or malignant hepatic and other organ lesions [2].

Due to the symptoms and progressive increase in the cyst’s size, the patient opted for surgical treatment and was admitted for laparoscopic marsupialization. Intraoperative evaluation revealed a non-infiltrative cystic lesion with a delicate plan of cleavage between the cyst wall and hepatic parenchyma. The cyst was aspirated, the fluid was sent for culture which came negative, and the wall was completely excised for pathologic examination.

**Figure 2:**
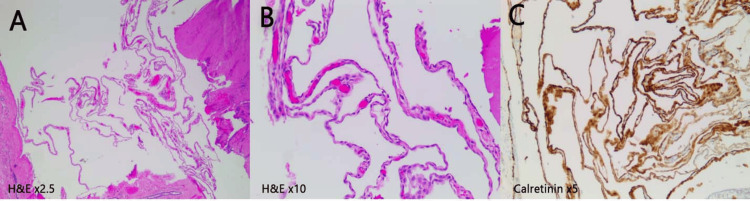
Microscopic picture of cyst wall Collapsed thin cyst wall (A) lined by one layer of flat to low cuboidal cells, (B) diffusely reactive for the mesothelial marker, and (C) calretinin X5

Grossly, the cyst wall was thin partly translucent, pale tan-pink collapsed membranous structure with fine vascularity. No discrete nodules or solid lesions were identified. Microscopic examination showed a thin fibro-connective cyst wall lined by a single layer of flat to low cuboidal cells consistent with benign mesothelium and supported by characteristic calretinin-positive immunohistochemical reactivity (Figure 2). The lack of cellular atypia or mitoses was consistent with the diagnosis of BCM.

The patient's postoperative period and follow-up evaluations showed complete recovery with no complications and resolution of the clinical symptoms.

## Discussion

Hepatic cysts encompass a wide spectrum of lesions including infection from different pathogens (pyogenic, amebic, echinococcal, etc.), pseudocysts, benign, premalignant, primary malignant, or metastatic tumors. Pseudocyst may be post-traumatic or from hematoma, seroma or biloma. Developmental cysts include lesions like simple liver cysts, polycystic liver disease, bile duct hamartomas, and Caroli disease. Premalignant and malignant cyst lesions include biliary cystadenoma, intraductal papillary neoplasm of the bile duct, and their malignant counterpart. Imaging studies (USG, CT scan, and MRI), in most of these lesions, display radiological features, which allow a non-invasive presumptive radiographic differential diagnosis. Hepatic BCM is the rarest among the long list of differential diagnoses of hepatic cysts.

BCMs, also known as peritoneal inclusion cysts, are nearly always reported in the pelvis of adult females. The nature of BCM is still controversial between neoplastic versus reactive processes. Neoplastic nature is suggested by the tendency to recur and the rare transformation to malignant mesothelioma [1]. The reactive process was attributed to frequent association with previous pelvic surgery, pelvic inflammatory disease, or endometriosis [3,4]. Most of the cases are asymptomatic or have non-specific symptoms. BCM in adult liver is extremely rare, and only one case was found in the English literature [5]. Imaging studies are helpful in limiting the radiographic differential diagnosis to the benign category of cystic lesions. Definitive characterization and cyst behavior are determined from histologic examination of the cyst wall and demonstration of its benign nature along with characteristic positive immunoreactivity staining pattern for mesothelial cell markers such as calretinin, cytokeratin, WT1, and/or D2-40. Surgical management is usually not necessary if there are no clinical or imaging concerns or asymptomatic. However, laparoscopic marsupialization or surgical excision may be recommended if the cyst is causing symptoms or complications or a definitive histologic characterization of the cyst is required to exclude underlying infection or malignancy [6,7].

## Conclusions

BCM is extremely rare in the liver with non-specific clinical presentation. Definitive cyst characterization and behavior are determined by histologic examination for demonstration of its benign mesothelial nature. Although BCM is the rarest among the long list of differential diagnoses of hepatic cysts, its identification in this rarely reported location is essential to avoid aggressive surgical treatment.
